# Balancing health and financial protection in health benefit package design

**DOI:** 10.1002/hec.4434

**Published:** 2021-10-08

**Authors:** Katherine T. Lofgren, David A. Watkins, Solomon T. Memirie, Joshua A. Salomon, Stéphane Verguet

**Affiliations:** ^1^ Interfaculty Initiative in Health Policy Harvard University Cambridge Massachusetts USA; ^2^ Division of General Internal Medicine University of Washington Seattle Washington USA; ^3^ Department of Global Health University of Washington Seattle Washington USA; ^4^ Department of Pediatrics and Child Health College of Health Sciences Addis Ababa University Addis Ababa Ethiopia; ^5^ Department of Global Public Health and Primary Health Care University of Bergen Bergen Norway; ^6^ Department of Health Policy Stanford University School of Medicine Stanford California USA; ^7^ Department of Global Health and Population Harvard T.H. Chan School of Public Health Boston Massachusetts USA

**Keywords:** benefit package design, constrained optimization, extended cost‐effectiveness analysis, financial risk protection, low‐ and middle‐income countries, priority setting, sustainable development goals, universal health coverage

## Abstract

Policymakers face difficult choices over which health interventions to publicly finance. We developed an approach to health benefits package design that accommodates explicit tradeoffs between improvements in health and provision of financial risk protection (FRP). We designed a mathematical optimization model to balance gains in health and FRP across candidate interventions when publicly financed. The optimal subset of interventions selected for inclusion was determined with bi‐criterion integer programming conditional on a budget constraint. The optimal set of interventions to publicly finance in a health benefits package varied according to whether the objective for optimization was population health benefits or FRP. When both objectives were considered jointly, the resulting optimal essential benefits package depended on the weights placed on the two objectives. In the Sustainable Development Goals era, smart spending toward universal health coverage is essential. Mathematical optimization provides a quantitative framework for policymakers to design health policies and select interventions that jointly prioritize multiple objectives with explicit financial constraints.

## 1 INTRODUCTION

The World Health Organization (WHO) has called for improved country support and guidance to achieve universal health coverage (UHC) through smarter spending strategies (World Health Organization, 2021). But what does “smarter spending” mean? Investments in publicly financed services and UHC must balance improvements to the scope of services covered, population access, and financial risk protection (FRP) for those seeking care (Roberts et al., 2015; World Health Organization, 2010). One way to communicate coverage of services and patient cost‐sharing transparently is to define the set of interventions that are fully or partially publicly financed in the form of an essential health benefits package (Verguet et al., 2021). Investing strategically toward UHC benefits from clear selection criteria and a robust decision framework that explicitly incorporates the preferences of the population. Coverage of services and cost sharing are distinct dimensions of benefit inclusion. Here, we consider the specific policy decision of what services to include in a national benefits package at no cost at the point of care.

One economic evaluation approach that is commonly used to determine the efficient allocation of resources within the health sector is cost‐effectiveness analysis (CEA) (Sanders et al., 2016). Cost‐effectiveness analysis aims to maximize the total improvements in population health that can be attained under a constrained budget. In practice, CEA is often conducted for a small number of interventions and alternative courses of action rather than for all possible interventions, and it relies on a specified value for the willingness to pay for a unit of health improvement (such as an averted disability‐adjusted life year [DALY]) as a benchmark to assess whether an intervention is worth the investment. Building on CEA, extended CEA (ECEA) disaggregates CEA outcomes across population subgroups (e.g., wealth quintiles) (Verguet et al., 2015, 2016). Extended CEA further departs from traditional CEA by reporting on both health and FRP benefits across socioeconomic groups using a dashboard approach. Multicriteria decision analysis is another framework that incorporates flexible social goals, but similar to ECEA, requires ad hoc aggregation of results, which may reduce interpretability (Baltussen et al., 2019; Morton & Lauer, 2017).

We propose mathematical optimization as a quantitative approach that is capable of explicitly incorporating multiple objectives to inform decision‐making in the health sector and the eventual design of health benefits packages in low‐ and middle‐income countries (LMICs). We focus on two major health system objectives, improving population health and FRP (Roberts et al., 2019), subject to a budget constraint. We illustrate our optimization approach with the case study of selected interventions in a low‐income country context. Recently, Karsu and Morton developed similar methods for trading off health and FRP benefits with multi‐objective optimization in the context of Malawi (Karsu & Morton, 2021). The work we present here builds on Karsu and Morton's contribution by focusing on the practical aspects of intervention selection, parameterization requirements and translation of WHO's UHC recommendations in the context of Ethiopia.

## 2 METHODS

Our analysis included two steps. First, we computed the health and FRP benefits expected for each candidate health intervention contingent on its inclusion in the benefits package (“Intervention‐level analysis”), assessed in comparison to a counterfactual in which that candidate intervention was excluded. Next, we applied constrained optimization to determine the optimal mix of candidate interventions given an objective (e.g., maximize health gains; maximize financial protection or both) and budget constraint (“Mathematical optimization analysis”).

As an illustration, we apply our optimization model to the case study of Ethiopia, a low‐income country in sub‐Saharan Africa with diverse epidemiologic needs. Ethiopia‐specific parameters were derived from the following sources: population estimates from the United Nations Population Division World Population Prospects (United Nations, 2017); national disease‐related mortality, incidence, and prevalence estimates from the 2017 Global Burden of Disease study (James et al., 2018; Roth et al., 2018), and intervention coverage from the 2016 Ethiopia Demographic and Health Survey (Central Statistical Agency [CSA] Ethiopia, 2017).

### 2.1 Intervention‐level analysis

We selected 20 interventions across 23 disease areas and age groups as an illustrative mix of candidate health interventions for benefits package consideration. The interventions were picked based on their relevance to WHO's UHC essential health service categories (Hogan et al., 2018). We also added a bundle of essential surgical procedures to our intervention choice set (Meara et al., 2015). Tables S1 and S2 in Supporting Information S1 provide details on both WHO's UHC categories and the specific interventions included in this analysis. All interventions were mapped to disease categories using the International Classification of Diseases 10. Intervention‐level input parameters were drawn from various sources (see Tables S3–S9 in Supporting Information S1). Labor and delivery interventions were broken down by signal function when possible with treatment effects for both neonatal and maternal burden targets (see Table S10 in Supporting Information S1) and then summed to account for the cumulative total health and financial benefits of labor and delivery intervention packages. Signal functions are evidence‐based practices used to measure if basic emergency obstetric care (BEmOC) or comprehensive emergency obstetric care (CEmOC) was available at delivery. For example, availability of uterotonic drugs (oxytocin) and parenteral anticonvulsants are two signal functions for access to BEmOC. Access to caesarean section and blood transfusion are the two signal functions that distinguish CEmOC from BEmOC.

**FIGURE 1:**
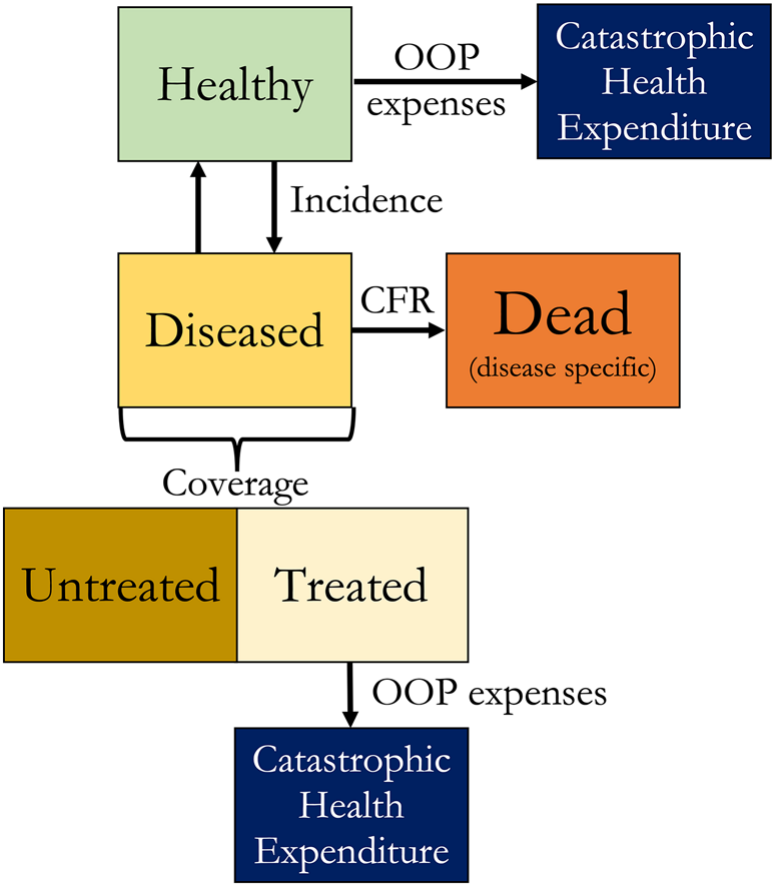
Conceptual disease‐state framework to model the health and financial risk protection benefits from inclusion of an intervention within a benefits package. Cases of catastrophic health expenditure occur when out‐of‐pocket (OOP) health‐related payments surpass a certain threshold of consumption expenditures. Patients are at risk of catastrophic health expenditure in the healthy state for preventive care and in the diseased state for curative care. CFR, case fatality ratio

We modeled each intervention's expected impact on population health and FRP using a simplified health‐state model (Figure 1). We assumed that inclusion of an intervention would eliminate out‐of‐pocket (OOP) payments for the intervention and would increase coverage by no more than 5% (i.e., an incremental coverage increase presumed to be achievable within current health system capacity). Interventions not included in the benefits package were assumed to maintain the baseline coverage and OOP payment levels in Ethiopia. Intervention unit cost estimates were obtained from the *Disease Control Priorities* 3rd edition (DCP3) for the World Bank's low‐income country grouping (Watkins et al., 2020). When unit cost data were not available from DCP3, we relied on estimates from the literature. National Health Accounts data complemented by the 2018 Noncommunicable Diseases and Injuries Commission Report informed the assumed percent of an intervention's unit cost that would be paid OOP by households at baseline (Federal Democratic Republic of Ethiopia, Ministry of Health, 2014, 2018); when unavailable for a specific disease category, the health sector average was used (approximately 34%; Table S5 in Supporting Information S1). To estimate the total cost to the government of including an intervention in the benefits package we assumed the government would cover the OOP household expenditures for the baseline coverage population and the full unit cost for all households newly seeking care (the incremental 5 percentage‐point coverage). All cost numbers were in 2016 USD. Some interventions include both a screening step and treatment conditional on a positive screen. For those interventions we estimated the percentage of households that received treatment conditional on screening and assumed those households incurred a unit cost specific to treatment in addition to a baseline screening cost (Table S6 in Supporting Information S1). In the model, individuals seeking preventive services are at risk of catastrophic health expenditures (CHEs, OOP expenses surpassing a certain threshold of consumption expenditures—a measure of lack of FRP) while in the healthy state in Figure 1. Individuals in the diseased state who access care and are treated are also exposed to a risk of CHE while receiving care based on the OOP expenses incurred.

Intervention health impact was modeled in two ways: via reduced disease incidence (directly or through risk factor control), and via reduced disease‐related fatality risks. In both instances, we quantified impact in terms of the number of deaths averted from an intervention coverage increase. Focusing on mortality outcomes undervalues diseases with high morbidity relative to mortality (such as mental health disorders) but was implemented for simplicity and due to data availability. The reduction in expected deaths from a coverage change (*C*_*i*_ to $C_{i}^{\prime }$) that acts on disease incidence was calculated as:

(1) $$\delta I_{i,\;x}=(C_{i}^{\prime }-C_{i})\times E_{i,x}\times A_{i,x}\times I_{x}\text{,}$$

(2) $$\delta D_{i,x}={\mathrm{CFR}}_{x}\times \delta I_{i,x}\text{,}$$

(3) $$\delta D_{i}=\sum_{x=1}^{N}\delta D_{x,i}\text{,}$$

where *E*_*i*,x_ is the effectiveness of intervention *i* on incidence of disease *x*, *A*_*i*,x_ is the share of disease *x* addressable (in the population) by intervention *i*, CFR_*x*_ is the case fatality ratio (CFR) of disease *x*, and *I*_*x*_ is baseline incidence of disease *x*. When an intervention directly modified mortality risks, the number of deaths averted was calculated based on the intervention impact on the current mortality burden, *D*_*x*_ (rather than incidence *I*_*x*_):

(4) $$\delta D_{i,x}=(C_{i}^{\prime }-C_{i})\times E_{i,x}\times A_{i,x}\times D_{x}\text{,}$$

where *E*_*i*,*x*_ is instead the treatment effectiveness of intervention *i* on deaths attributable to disease *x*, and *D*_*x*_ is baseline disease‐related mortality.

Intervention coverage increases today can produce both immediate and future health benefits and financial consequences. For example, when a child is treated with antibiotics for meningitis, both health benefits and OOP expenditures happen immediately. By contrast, when the coverage of screening and control of diabetes increases, health benefits are typically delayed while the financial consequences may occur both today (control) and in the future (through the treatment of diabetes complications). For ease of exposition, we considered a “steady state” system in which coverage increases have immediate health and financial benefits. We also considered a scenario with benefit and cost consequence time lags based on the natural history of each disease for a cohort of present‐day individuals. Where delayed effects were relevant, we binned interventions into either short‐, medium‐, or long‐term categories with time delays for the health and FRP benefits of 5, 10, and 20 years, respectively (and with immediate costs). Rotavirus vaccination in newborns is one example of a short‐term delayed intervention; screening and control of hypertension in 50–69 year‐olds is categorized as a medium‐term delayed intervention; and human papillomavirus vaccination of 12‐year‐old females is the only intervention categorized in the 20‐year delay category (Table S3 in Supporting Information S1).

Inclusion of an intervention in the benefits package was assumed to produce FRP (i.e., reduction in estimated CHE cases) through two channels. First, we assumed that inclusion of services in the package eliminated all OOP direct medical costs associated with those services. We considered OOP direct medical costs only, which is consistent with the computations of CHE estimates routinely provided by WHO and the World Bank (Wagstaff et al., 2018; World Health Organization & World Bank, 2017). For example, if the benefits package included rotavirus immunization, households would no longer incur the baseline OOP immunization expenses. We refer to this channel as “primary” FRP. In addition, covered services may also provide “secondary” FRP. With expanded coverage of rotavirus immunization, more children are vaccinated, reducing the likelihood of severe rotavirus diarrhea in those same children. The reduced need for diarrheal treatment (e.g., oral rehydration solution) and associated OOP expenses would be averted if rotavirus were included in the benefits package. We estimated both primary and secondary FRP benefits per intervention by mapping all interventions temporally by disease target, that is, rotavirus is an “earlier” intervention compared to oral rehydration solution for the treatment of diarrheal disease (Table S4 in Supporting Information S1).

We quantified the FRP benefits in terms of CHE cases averted when each intervention is publicly financed through a benefits package, compared to a counterfactual in which that intervention is excluded. Health expenditures were deemed “catastrophic” when direct household OOP health expenditures would exceed a pre‐defined threshold (*t*_CHE_) of a household's per capita total consumption expenditures (*y*_*h*_). We used the threshold of 10% of per capita household consumption in our main analysis, and included a sensitivity analysis with a threshold of 25% (as in Wagstaff et al., 2018). CHE cases averted were calculated for both households who sought care at baseline (and incurred OOP expenses) and households newly seeking care conditional on an intervention inclusion in a benefits package. CHE cases averted are a function of the change in OOP expenditures as a percent of household per capita total consumption expenditures (Equations (5), (6), (7) below).

(5) $$T_{i,x}=C_{i}^{\prime }\times A_{i,x}\times {\mathrm{POP}}_{i,x}\text{,}$$

(6) $$\delta H_{i}=\sum_{x=1}^{N}T_{i,x}\text{,}$$

(7) $$\delta {\mathrm{CHE}}_{i}=\sum_{\;h\;\in \;H_{i}^{\prime }}\{\begin{array}{ll} {\mathrm{OOP}}_{i}>t_{\mathrm{CHE}}\times y_{h}\; & 1\\ \mathrm{else} & 0 \end{array}\;\text{.}$$

where *T*_*i*,x_ is the number of individuals treated conditional on an intervention inclusion in a benefits package (and assumed shift to coverage $C_{i}^{\prime }$). *δH*_*i*_ are the covered households, and POP_*i*,*x*_ is the target population for each intervention (which can be the general population, the incident or prevalent populations, depending on the disease).

For health interventions that target multiple disease categories (e.g., the basic surgical package targets road traffic injuries and falls), the ensemble $H_{i}^{\prime }$ included households across all relevant disease targets. Households newly seeking care (due to increased coverage) were counted as CHE cases averted if the baseline OOP expenditures (now waived) for the service constituted expenditures above the 10% threshold of consumption expenditures. Since we assumed inclusion of an intervention eliminates all OOP expenditures, we counted all the associated expected CHE cases as CHE cases averted when the intervention is publicly financed. When relevant, secondary FRP benefits were estimated as decreased demand for downstream care:

(8) $$\delta H_{i,j}=\sum_{x=1}^{N}\delta I_{i,x}\times C_{j}\times A_{j,x}\text{,}$$

where *i* indexes the intervention under consideration for benefit package inclusion and *j* indexes downstream care relevant to the same disease class *x*.

Per intervention, estimates of deaths averted and CHE cases averted were computed including upper and lower bound estimates based on uncertainty available for the treatment effect and the disease burden (deaths, incidence, and prevalence estimates). We conducted scenario analyses to estimate optimistic and pessimistic outcomes (see section 14 “Scenario cases” in Supporting Information S1). Briefly, we took the set of lower, mean, and upper bounds for disease burden (deaths, incidence, and prevalence) in combination with the lower, mean, and upper bounds of each treatment effect estimate (9 total combinations). Finally, we extracted the highest and lowest deaths averted and CHE cases averted outcomes for the optimistic and pessimistic scenarios.

We approximated the distribution of household consumption expenditures per capita using a gamma distribution where scale and shape parameters are a function of the per person total household expenditures and Gini coefficient for Ethiopia (see section 5 “Household consumption distribution” in Supporting Information S1). We assumed that household demand for each intervention is constant across all household income levels: this is a strong assumption which was taken due to lack of appropriate available data. The total cost (denoted TC_*i*_) to fund each intervention was estimated as the intervention unit cost times the new treatment population (i.e., the ensemble of households $H_{i}^{\prime }$). All intervention unit costs and OOP costs are summarized in Tables S5 and S6 of Supporting Information S1. To incorporate uncertainty in unit costs, we considered optimistic and pessimistic scenarios as ±50% the base‐case unit costs as we lacked empirical uncertainty ranges for costs.

### 2.2 Mathematical optimization analysis

We used integer programming to identify the optimal subset of interventions to include in the benefits package under a given budgetary constraint, denoted *B*. First, we optimized on a single objective, ${\text{Obj}}_{i}^{1}\text{,}$ that was defined as either deaths averted (*δD*_*i*_, per intervention *i*) or CHE cases averted (*δ*CHE_*i*_, per intervention *i*). Second, we also performed a bi‐criterion optimization by optimizing on one objective, ${\text{Obj}}_{i}^{1}\text{,}$ and incorporating the second objective, ${\text{Obj}}_{i}^{2}$, as a constraint. For instance, if we maximized deaths averted, we included an additional constraint that CHE cases averted exceeded a given constant *K*. By varying *K*, a Pareto efficiency frontier was generated. When *K* equals 0, the outcome is at least as good as the single objective optimization of ${\text{Obj}}_{i}^{1}$. When *K* equals the maximum possible solution for ${\text{Obj}}_{i}^{2}$, there is forced consistency with the single objective optimization of ${\text{Obj}}_{i}^{2}$. In this analysis, *K* ranged from 0 to 115,747 for CHE cases averted and from 0 to 6010 for deaths averted. Our bi‐criterion optimization is expressed as:

(9) $$\mathrm{Max}\sum_{i=1}^{N}{\text{Obj}}_{i}^{1}\times Z_{i}\text{,}$$

subject to:

$$\sum_{i=1}^{N}{\mathrm{Obj}}_{i}^{2}\times Z_{i}\geq K\text{,}$$

$$\sum_{i=1}^{\;N}{\mathrm{TC}}_{i}\times Z_{i}\leq B\text{,}$$

$$Z_{i}\;\in \{0,1\},i=1,\;2,...,N\text{,}$$

where *Z*_*i*_ is a binary decision variable for inclusion of intervention *i* (*Z*_*i*_ equals 0 or 1), *K* is a positive integer that captures the increase in the second objective, B is the budgetary constraint, and TC_*i*_ is the cost of including intervention *i* (at no cost to treated individuals). We considered a range of constraint values (*K*) for the second objective that covered the entire feasible funding space to generate the Pareto efficiency frontier; and *K* was incrementally changed by single units of either deaths averted or CHE cases averted. The technical appendix of Karsu and Morton (Karsu & Morton, 2021) provides a detailed explanation for implementation of the epsilon constraint method which can efficiently modify the value of K iteratively (see also Ehrgott, 2005). The analysis used R statistical software (version 3.5.0; www.r‐project.org). All data, code, and result files are available on request.

## 3 RESULTS

### 3.1 Intervention‐level analysis

**FIGURE 2:**
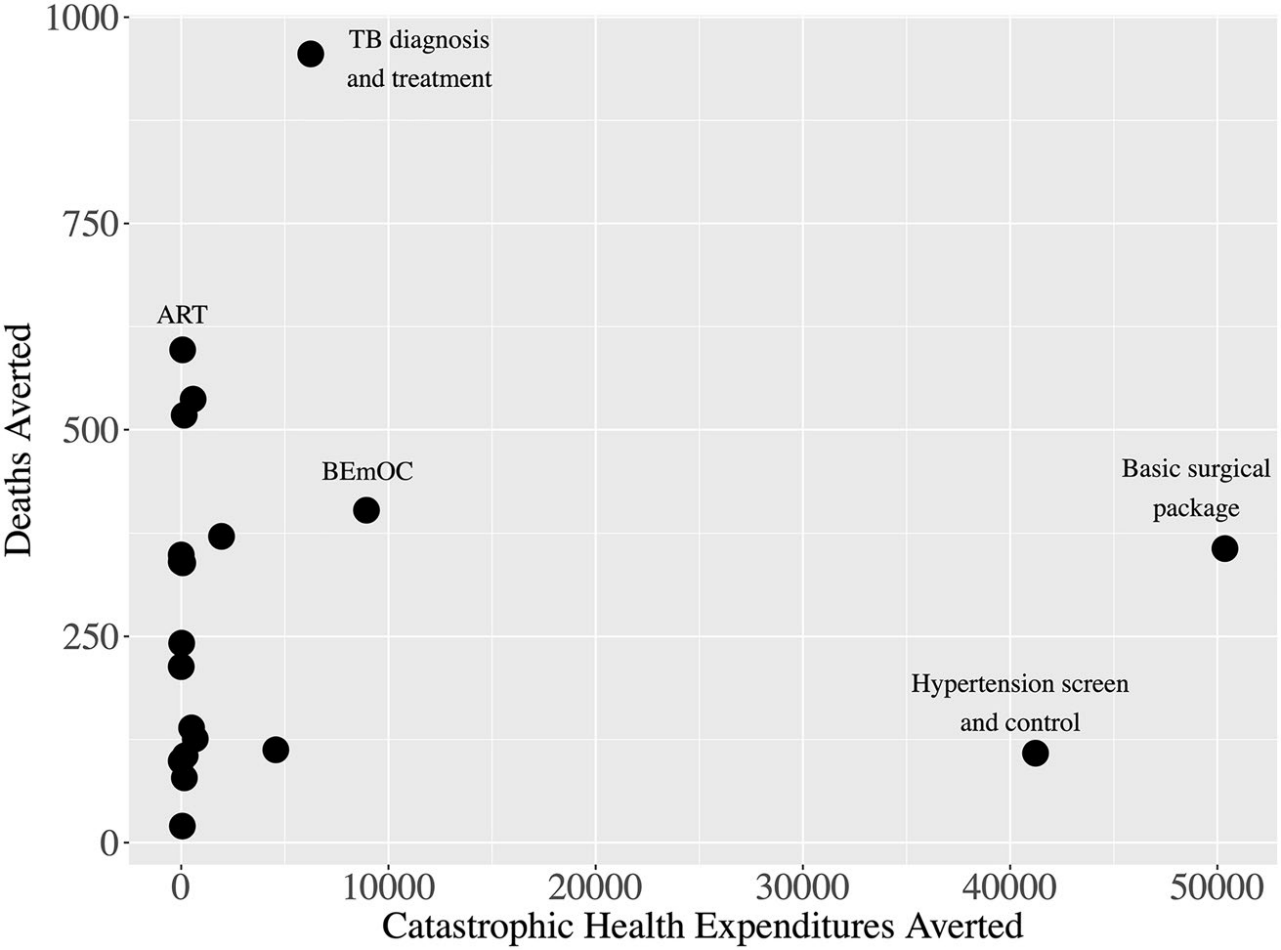
Intervention‐level expected deaths averted and cases of catastrophic health expenditures (CHEs) averted with a coverage expansion of 5 percentage points compared to baseline intervention coverage levels. ART, antiretroviral treatment; BEmOC, basic emergency obstetric care; TB, tuberculosis

Figure 2 shows the intervention‐level results for deaths averted and CHE cases averted when an intervention was included in the benefits package free of all OOP expenditures (Table S12 in Supporting Information S1 lists upper and lower bound estimates). There are few interventions that are clear priorities across both outcomes. Further, Figure 2 does not include information on the cost of including each service in the benefits package. A package of basic surgical services and screening and control of hypertension are both high‐value interventions as measured by the number of CHE cases averted. However, these are also the two most expensive interventions, with price tags of 21 and 15 million USD respectively.

### 3.2 Mathematical optimization analysis

**FIGURE 3:**
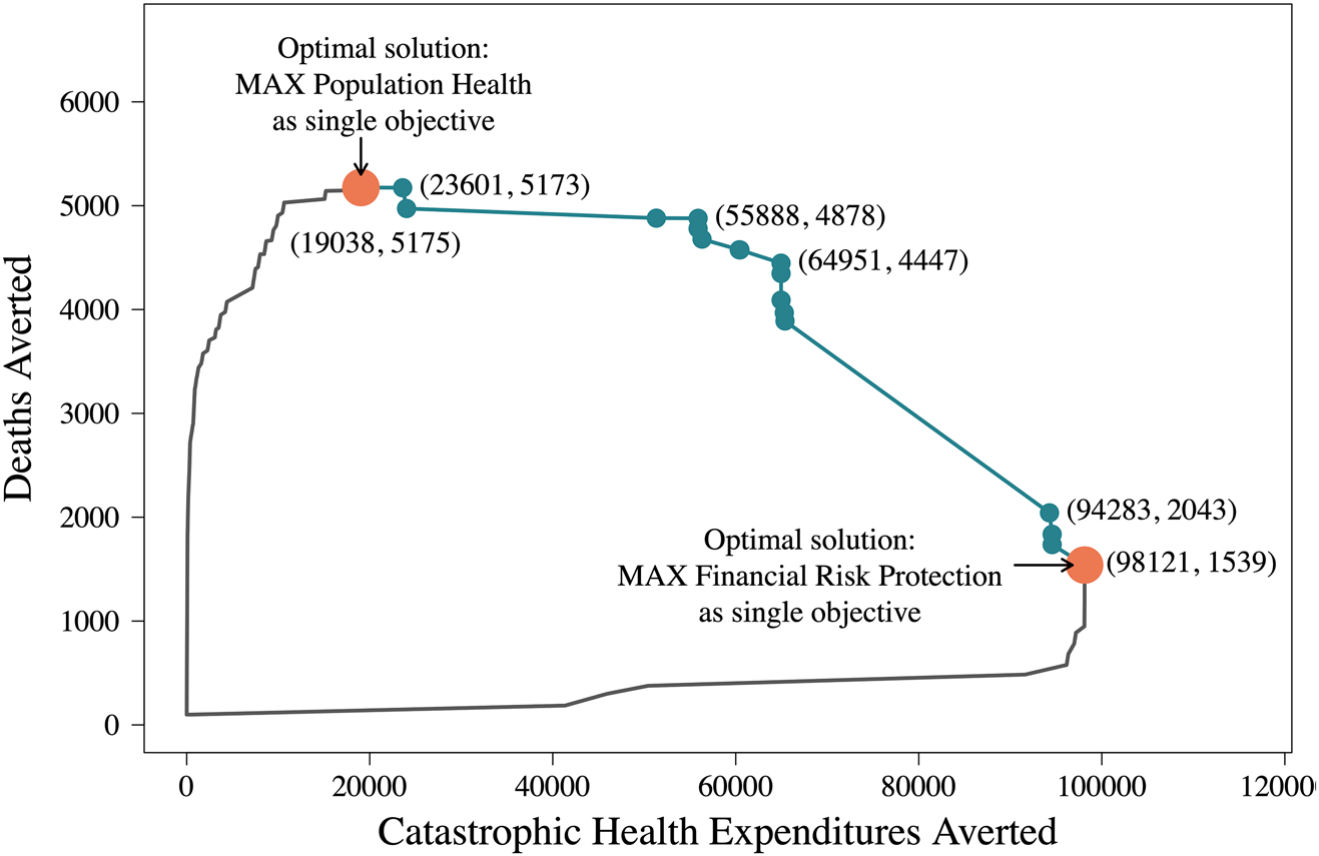
Benefit package solutions and Pareto efficiency frontier, based on the objectives of maximizing population health (deaths averted) and maximizing financial risk protection (catastrophic health expenditures cases averted), with a 40 million USD budget constraint. The orange dots represent the optimal solutions when either population health or financial risk protection (FRP) are considered as single objectives. The green line is the Pareto efficiency frontier, and each green point along the frontier is a possible benefits package that requires specific tradeoffs between health and FRP outcomes

We first analyzed results at a budget constraint of 40 million USD as the total budgetary constraint of a potential benefits package (zero‐base). As context, the total health expenditures with government financing in Ethiopia was approximately 700 million USD in 2016/2017 (Federal Democratic Republic of Ethiopia, Ministry of Health, 2019). Figure 3 shows the fundable space (area within the lines) and the two single‐objective optimal solutions (large orange points) to maximize either deaths averted or CHE cases averted subject to the budget constraint. The green line connecting the two orange points represents the Pareto efficiency frontier. To move from one extreme to the other, specific tradeoffs in the two outcomes of interests, population health and FRP, are required. The green dots along the frontier represent specific benefit packages that are affordable at the budget of 40 million USD.

**FIGURE 4:**
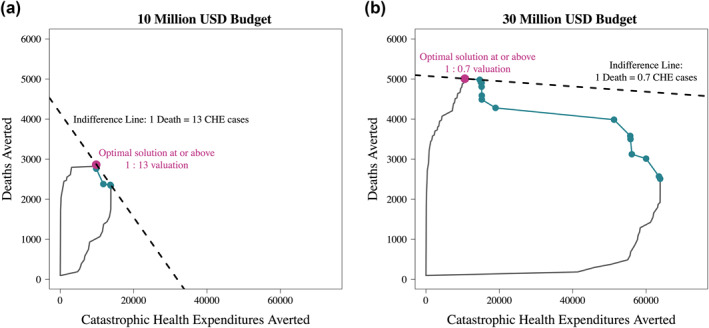
Benefit package solutions and Pareto efficiency frontier, based on the objectives of maximizing population health (deaths averted) and maximizing financial risk protection (cases of catastrophic health expenditures averted), under a budget constraint of 10 (a) and 30 million USD (b). The purple point is the optimal solution when deaths averted are as important as the indifference equation and dotted line in each graph [1:13 in (a) and 1:0.7 in (b)]. The green dots are possible solutions along the Pareto frontier (green line)

Although multiple points along the Pareto frontier may, by definition, be optimal, these points do not represent equivalent solutions. As population health becomes more important relative to FRP, the optimal solution shifts toward the solution under the single objective optimization until the bi‐objective and population health single‐objective solutions converge. We highlight this situation under budget constraints of 10 and 30 million USD (Figure 4). The convergence of bi‐objective optimization and population health single‐objective optimization depends on the shape (curvature) of the Pareto frontier. When the budget is 10 million USD, solutions converge when 1 death averted is equivalent to at least 13 CHE cases averted (pink dot in Figure 4a); for a 30 million USD budget, the convergence occurs when 1 death averted is equivalent to at least 0.7 CHE cases averted (pink dot in Figure 4b). Once the solutions converge, further increases in the value of deaths averted compared to CHE cases averted have no effect on the optimization solution. For example, in Figure 4a (10 million USD budget), if one death averted is weighted equivalent to 14 or more CHE cases averted, the solution remains the same as when 1 death averted is weighed equivalent to 13 CHE cases averted. If policymaker relative valuations for deaths averted compared to CHE cases averted are elicited, their preferences will only influence the optimal benefits package when CHE cases are valued more than the number of CHE cases averted at the convergence point. In Figure 4b for example, as long as policymakers agree that one death averted is worth at least one CHE case averted, there is no need to elicit more precise preferences: a bi‐criterion optimization solution will be equivalent to a single objective optimization solution to minimize deaths averted with no consideration of CHE cases averted because the convergence occurs at one death averted equivalent to 0.7 CHE cases averted. The dotted lines are indifference curves whose slopes are consistent with the relative value of deaths averted versus CHE cases averted required for the bi‐criterion and single‐objective optimization solutions to converge.

**FIGURE 5:**
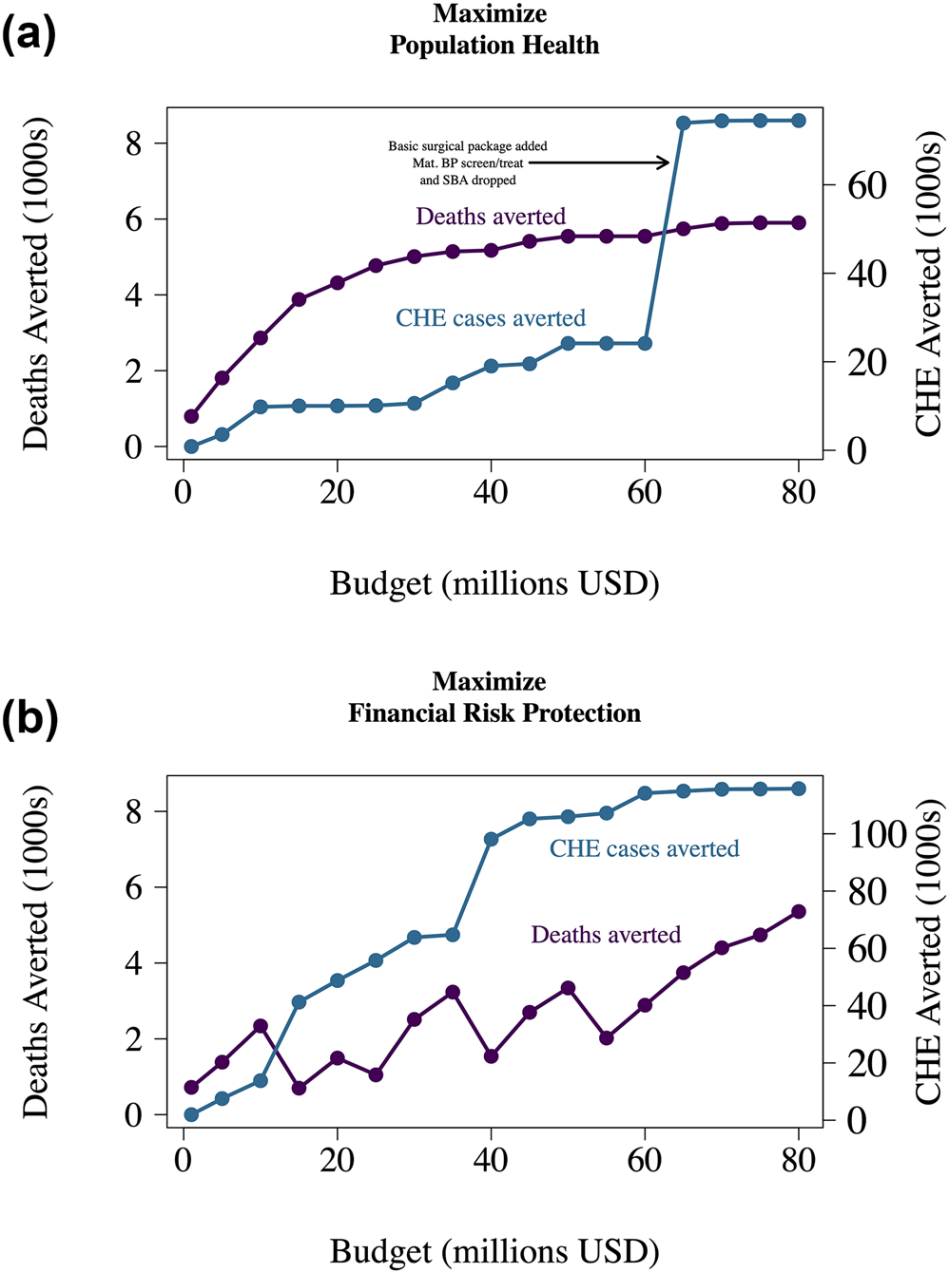
Optimal benefit package solutions based on single‐objective optimizations of either maximizing population health (deaths averted) or financial risk protection (cases of catastrophic health expenditures [CHEs] averted), under a budget expansion from 1 to 80 million USD. Each panel is a single‐objective optimization of either deaths averted (a) or cases of CHEs averted (b). Results for CHE cases averted in (a) and deaths averted in (b) are plotted based on the optimal benefit package at each budget constraint value but they were not included in the single‐objective optimization as an explicit objective or a constraint. Mat. BP screen/treat, maternal blood pressure screening and treatment with calcium supplementation; SBA, skilled birth attendance

Setting a larger budget enables greater improvements in the two outcomes of population health (deaths averted) and FRP (CHE cases averted) generally. This is illustrated by the expansion of the feasible funding space between Figure 4a and 4b (toward the upper right corner; Figure 4). However, in a single‐objective optimization, the auxiliary outcome (either deaths averted or CHE cases averted) does not necessarily increase monotonically as budget increases. Figure 5 plots the expected changes in both outcomes (deaths and CHE cases averted) when benefit package design is approached as a single‐objective optimization problem. Incremental changes in budget at times may decrease the objective not accounted for in the optimization (i.e., the auxiliary outcome). Particularly for the single‐objective optimization of CHE cases averted (Figure 5b), we observe sporadic decreases in deaths averted (the outcome not accounted for in the optimization) for the selected benefits package. Figure 5a,b represents the “extreme” solutions (single‐objective optimization solutions of deaths averted and CHE cases averted, respectively) across a range of budgets including those in Figure 4. Given that the bi‐criterion optimization converges to the single‐optimization solution at relatively low rates of substitution of CHE cases averted for deaths, Figure 5a shows relatively stable results (with only slight decreases in CHE cases averted at incremental budget increases) compared to Figure 5b.

Another feature of the single‐objective optimization results is that some budget increases can lead to large incremental outcome changes. With incremental budget increases, there are instances where a high‐value intervention in one or more outcome becomes newly affordable.

For example, Figure 5a highlights the importance of the budget increases from 60 to 65 million USD: with the 5 million USD increase, a basic surgical package becomes affordable, an intervention that is estimated to avert 50,364 CHE cases (and 356 deaths) when included. While a basic surgical package becomes affordable at a budget of 65 million, both maternal blood pressure screening and control and skilled birth attendance are simultaneously dropped. High‐cost, high‐value interventions can come in and out of optimal benefit package solutions as the budget incrementally changes. Additional results and sensitivity analyses are available in the Supporting Information S1 that explore considerations including parameter uncertainty (using upper and lower bounds for intervention efficacy, target population size, incidence, CFR, mortality burden, and unit costs), bundled intervention funding, and a catastrophic threshold of 25% (instead of 10%) (see Figures S1–S3 in Supporting Information S1). Figure S1 in Supporting Information S1 shows the uncertainty in both outcomes (deaths averted and CHE cases averted) for budgets up to 80 million USD for the single‐objective optimizations (the ones reported in Figure 5). When the benefit package is optimized for CHE cases using a 25% catastrophic threshold (see Figure S2 in Supporting Information S1), the level of CHE cases averted remains relatively stable. When interventions are bundled, incremental budget changes can be an opportunity to fund an entire bundle that was not previously affordable (see Figure S3 in Supporting Information S1). Given that bundled interventions (e.g., childhood immunizations) can reflect important real‐world decision scenarios, understanding the resulting outcomes across a range of budgetary levels is critical to identify high‐value incremental budget increases for improved population health and FRP.

## 4 DISCUSSION

Mathematical optimization allows analysts to simultaneously specify multiple constraints and objectives that matter to decision‐makers. Particularly in the context of publicly financed UHC, mathematical optimization can incorporate a set of criteria and outcomes of interest. We explicitly considered here the two major health system objectives of improving population health and FRP within an optimization approach which we illustrated with a case study of Ethiopia. Our findings demonstrate substantial tradeoffs between health maximization (captured by maximizing deaths averted) and FRP maximization (captured by maximizing CHE cases averted). Multiple “optimal” benefit packages could be selected for public financing depending on the relative local policy importance given to population health compared to FRP.

Essential benefit packages (EBPs) commonly cover both preventive and curative services. Preventive care has the potential to avert intensive and costly curative interventions but also requires coverage across large populations. By contrast, focusing on curative care may enhance care‐seeking and utilization for those most in need but often has high cost per patient treated. Our analysis demonstrates how mathematical modeling in tandem with optimization can formally account for service‐type tradeoffs by temporally mapping interventions and accounting for both the health and FRP benefits of each candidate intervention. However, selecting what interventions are being prioritized at different budget levels depends on numerous factors with multiple sophisticated interactions. Hence, it remains difficult to interpret here prioritization differences across distinct budget level constraints in a generalizable manner, a limitation (at this preliminary exposition stage) of this complex type of analysis.

Our analysis has a number of limitations. First, in the development of our optimization model, we made several simplifying assumptions: we only considered two major objectives of health systems (health and FRP maximization). We did not formally account for health disparities (e.g., distribution of health outcomes across socioeconomic groups) or priority to the worse‐off or special disadvantaged groups. We also limited our health outcome to deaths averted omitting DALY estimation (see, e.g., Karsu & Morton, 2021 for the use of DALYs as a health outcome measure). Deaths averted, as a health outcome, was chosen for local policy relevance (e.g., high mortality levels in Ethiopia), simplicity of exposition, and due to paucity of data. However, future work could consider DALYs or other measures (like quality‐adjusted life years) that capture both morbidity and mortality consequences of interventions. We also did not consider supply side constraints such as human resources availability or facility capacity (Smith & Yip, 2016). More generally, many additional constraints could be embedded into our optimization problem (see section 13 “Additional optimization constraints” in Supporting Information S1). Second, in our Ethiopia example, we relied on a variety of input parameters drawn from heterogeneous data sources of varying quality. Additionally, we only considered direct medical expenditures. Yet, there is evidence that indirect costs (e.g., time losses and wages lost with the onset of illness) and transportation costs can amount to a large portion of overall illness‐induced expenditures for households. Due to lack of available data and to be consistent with the way CHE cases are being estimated by the World Bank and WHO (Wagstaff et al., 2018; World Health Organization & World Bank, 2017), we only considered direct OOP medical costs, which might underestimate the potential FRP benefits of certain interventions. Third, we assumed constant household demand for interventions regardless of household income, when in reality wealthier households may be more likely to seek care at baseline (CSA Ethiopia, 2017). This assumption may overlook important high‐value interventions for low‐income populations in particular. If a benefits package was designed specifically for the poor, it would be important to modify the parameters and income distribution used in this analysis to reflect heterogeneities in disease burden, healthcare utilization, etc. across income levels. Fourth, our analysis relied on a simplified static disease modeling framework to estimate the impact of intervention coverage increases on mortality and financial outcomes. That design does not capture secondary effects and dynamic transmission pathways or fully incorporate the natural history of diseases. Rather, our disease modeling was meant to be illustrative as our analysis focused on mathematical optimization with two distinct objectives to select health interventions for priority setting, rather than detailed epidemiological modeling. We acknowledge that EBP design is meant to be transparent, participatory, and accountable and that quantitative analysis is one input in the process. At the same time, people agree that CEA is important to EBP design and that planners need information on costs and budget constraints. Here, we take this acknowledged need one step further and quantify the FRP dimension of UHC so that it can be explicitly integrated into existing optimization approaches, which mostly look at population health only.

This work is grounded in a growing literature that calls for or applies mathematical optimization and operations research methods to resource allocation problems in health policy (examples include Crown et al., 2017; Epstein et al., 2007; Kerr et al., 2015; Ochalek et al., 2020; Smith & Yip, 2016; Verguet, 2013; Wilson & Blower, 2006; and most recently Karsu & Morton, 2021). With the move toward UHC as a core component of the SDGs, revising and updating national essential health benefit packages will become an increasingly important policy milestone in many LMICs (Verguet et al., 2021). At the local level, a critical and daunting task is the technical feasibility of benefits package revision when hundreds of interventions are under consideration. While the approach presented here does not eliminate the need to elicit the relative valuation of health benefits compared to FRP benefits, it nonetheless can help communicate how relative valuations will modify the optimal solution as well as identify budgets where a single‐objective solution may be sufficient to capture high value across multiple dimensions. For example, in the 10 million USD budget case, as long as there is agreement that one death averted is as or more valuable than thirteen CHE cases averted, the optimal solution will always be the optimal package highlighted in Figure 4a (i.e., the single‐objective optimization package that maximizes population health). If that is the fixed budget and there is agreement that one death averted is worth at least thirteen CHE cases averted, estimating the relative valuation of deaths averted to CHE cases averted becomes irrelevant for the package design. Our analysis focused on a small set of interventions to be provided at no OOP cost to the entire population, with the choice set of candidate interventions first pre‐screened based on cost‐effectiveness and other criteria. In reality, benefit package design analysis could require consideration of scores of hundreds of candidate interventions. In the future, one will need to increase the number of interventions analysed and to develop more sophisticated modeling approaches, including costs. Methods that can transparently inform the design of such packages of publicly financed interventions have the potential to initiate national and local discussions on how to balance the many—and sometimes competing—objectives of UHC including: efficiency, FRP, and equity. In the Sustainable Development Goals era, constrained optimization methods can meaningfully contribute to public financing discussions on how to spend money wisely.

## CONFLICT OF INTEREST

All authors declare no conflicts of interest.

## Supporting information

Supplementary Material

## Data Availability

All code and underlying data are available on GitHub.
