# Supplementary material for: Balancing health and financial protection in health benefit package design
Source: Health Econ. 2021 Oct 8;30(12):3236–47. doi: 10.1002/hec.4434 (PMC9293346; doi:10.1002/hec.4434)
Supplement: Supplementary file 1 — Supplementary Material [file HEC-30-3236-s001.docx]

**Supplementary Webappendix:**

**Balancing Health and Financial Protection in Health Benefit Package Design**

Katherine T. Lofgren, David A. Watkins, Solomon T. Memirie, Joshua A. Salomon, Stéphane Verguet

This document provides supplementary information to the main paper. Each of the 14 sections are listed below.

Appendix Section 1. The World Health Organization tracking of essential health services

Appendix Section 2. Summary of the intervention choice set

Appendix Section 3. Intervention time lags for discounting scenarios

Appendix Section 4. Temporal placement of interventions within the choice set

Appendix Section 5. Household consumption distribution

Appendix Section 6. Unit costs and out-of-pocket expenditures

Appendix Section 7. Intervention baseline coverage

Appendix Section 8. Disease burden

Appendix Section 9. Intervention treatment effects

Appendix Section 10. Labor and delivery care specific considerations

Appendix Section 11. Supplementary results

Appendix Section 12. Net present value optimization

Appendix Section 13. Additional optimization constraints

Appendix Section 14. Scenario cases

# Appendix Section 1. The World Health Organization tracking of essential health services

The World Health Organization (WHO) has proposed a series of indicators to measure and track universal health coverage (UHC) (Hogan et al., 2018). The categories used to track UHC have been incorporated into this analysis when possible. Appendix Table S1 summarizes all the categories included in the WHO essential health services index. Bolded categories have been included in our analysis.

**Appendix Table S1:** WHO universal health coverage indicators

^1^ UHC indicators directly addressed in our study.

# Appendix Section 2. Summary of intervention choice set

The interventions included in our analysis are intended to be representative across age- and sex-specific populations as well as across both communicable and non-communicable disease categories. For graphical purposes, abbreviated titles are often used. Appendix Table S2 summarizes both the full and short names of interventions as well as the target age- and sex-specific populations for each service.

**Appendix Table S2:** Intervention choice set with age- and sex-specific target subpopulations

Note: TB = tuberculosis, ART = antiretroviral therapy, HPV = Human papillomavirus, DVI = direct vaginal inspection, IHD = ischemic heart disease.

# Appendix Section 3. Intervention time lags for discounting scenarios

Increased coverage of an intervention today can lead to immediate benefits for some populations – like surgical services for trauma patients. In other cases, the benefits can be delayed. For example, controlling risk factors like blood pressure modifies the risk of ischemic heart disease years into the future. Appendix Table S3 lists all the interventions in our choice set as well as the assumed average time delays for the health benefits to manifest. We use these approximate time delay estimates (t) to optimize a benefits package where all health benefits ($D_{i}^{'}$) and secondary downstream financial risks (captured by estimated cases of catastrophic health expenditures (CHE)) from future health care use were discounted to net present value benefits (${NPV(D}_{i}^{'}$), ${NPV(CHE}_{i}^{'})$) with a 3% annual discount rate:

${NPV(\delta D}_{i})= \frac{{\delta D}_{i}}{{1.03}^{t}}$ .

${NPV(\delta CHE}_{i})= {\delta CHE}_{primary,i}+ \frac{{\delta CHE}_{secondary,i}}{{1.03}^{t}}$ .

**Appendix Table S3:** Average time delays for intervention benefits.

Note: TB = tuberculosis, ART = antiretroviral therapy, HPV = human papillomavirus, IHD = ischemic heart disease.

# Appendix Section 4. Temporal placement of interventions within the choice set

All interventions included in the analysis were mapped to relevant disease- and age-specific categories as well as temporally placed within the cascade of care. For example, in the case of diarrheal disease in under-five year-olds, two interventions were included in the analysis: rotavirus vaccine and oral rehydration solution (ORS). Because the rotavirus vaccine targets a population of infants, it occurs earlier in the cascade of care. This implies that increasing coverage of the rotavirus vaccine not only would lead to primary benefits, but also would modify demand for downstream care (like ORS) due to reduced disease cases. Appendix Table S4 lists all included disease categories and the temporal placements of relevant interventions.

**Appendix Table S4:** Intervention temporal placement, by disease target

Note: TB = Tuberculosis, ART = antiretroviral therapy, HPV = human papillomavirus, IHD = ischemic heart disease.

# Appendix Section 5. Household consumption distribution

We used a mean estimate of per person total household consumption expenditures accounting for the distribution of household size corresponding to that of Ethiopia (which was available from the 2015-2016 Ethiopian Household Consumption – Expenditure (HCE) survey; see Table B1.1, page 143) (The Federal Democratic Republic of Ethiopia, 2018).

Converting 9,626.78 Birr to USD based on the exchange rate on July 2nd, 2016 (21.7 Birr to 1 USD) the estimate of average per person household consumption was set to 443.63 USD annually.

The mean household consumption as well as the Gini coefficient was then used to create a household consumption distribution using a gamma distribution building on (Salem & Mount, 1974).

The R code to implement this strategy is:

fgamma <- function(phi){gini-(1/(phi*4^phi))*1/beta(phi,phi + 1)}

phi <- uniroot(fgamma,lower=0.000001,upper=100)$root

bea <- (1/phi)*(ave_hh_consumption)

gen.draws <- function(y){rgamma(y,shape=phi,scale=beta)}

draws <- gen.draws(total.draws)

Where **total.draws** is user-defined and **ave_hh_consumption** is user defined and equal to **443.63** **USD** for this analysis.

# Appendix Section 6. Unit costs and out-of-pocket expenditures

Unit costs for interventions were obtained from the *Disease Control Priorities* Third Edition (DCP3, www.dcp-3.org). In some instances, interventions included in the analysis were not readily available from DCP3. When needed, unit costs were generated using other sources including from the published literature to be consistent with DCP3 results generated by Watkins et al. (Anh et al., 2010; Watkins et al., 2020). Out-of-pocket (OOP) expenditure estimates were estimated using data from Ethiopia’s 5^th^ National Health Accounts and the Noncommunicable Diseases and Injuries (NCDI) Commission Report (*Ethiopia’s 5th National Health Accounts*, 2014; *Ethiopia’s NCDI Commission Report*, 2018). These estimates included percentages of health expenditures classified as out of pocket typically by type of services and disease categories. When no information specific to a health intervention class included was available, the published literature was used when available or alternatively the average OOP health expenditure estimate from the National Health Accounts (i.e. 34%) was used (*Ethiopia Health Accounts, 2013/2014*, 2017). Appendix Table S5 summarizes the unit and OOP costs used in our analysis.

**Appendix Table S5:** Intervention unit costs and corresponding out-of-pocket (OOP) costs.

Note: TB = Tuberculosis, ART = antiretroviral therapy, HPV = human papillomavirus, IHD = ischemic heart disease.

# In some cases, interventions include both a screening step and a treatment step conditional on screening results. Appendix Table S6 breaks down the probability that a patient in our model passes from the screening step to the treatment as well as the unit costs specific to screening and treatment. The average unit costs of these interventions (listed above in Appendix Table S5) are used to estimate the total cost of including the intervention in the benefit package. The screening and treatment specific unit costs as well as the probability of a screened patient being treated are used to estimate cases of catastrophic health expenditures. This disaggregation is important because treatment is typically significantly more expensive than screening. For maternal blood pressure screening and TB screening, we limit those screened to presumptive cases and assume a 100% treatment rate of those screened.

**Appendix Table S6:** Screening and conditional treatment intervention unit costs.

# Appendix Section 7. Intervention baseline coverage

Baseline coverage levels for each intervention were drawn from surveys including Ethiopia’s Demographic Health Survey and the published literature (Admasu et al., 2011; *Ethiopia Demographic and Health Survey 2016*, 2016; *Ethiopia Mini Demographic and Health Survey Key Indicators*, 2019; *Ethiopia STEPS Report on Risk Factors for Chronic Non-Communicable Diseases and Prevalence of Selected NCDs*, 2016; *OneHealth Tool*, 2017; *UNAIDS 2019 Estimates*, 2018; *WHO Tuberculosis Profile: Ethiopia (Shiny App)*, 2020; Tolla et al., 2016). Appendix Table S7 summarizes the baseline coverage levels used in the analysis for each intervention as well as the source(s) of the information.

**Appendix Table S7:** Baseline population coverage, by intervention

Note: TB = Tuberculosis, ART = antiretroviral therapy, HPV = human papillomavirus, IHD = ischemic heart disease.

# Appendix Section 8. Disease burden

Incidence, prevalence, and deaths were obtained by downloading age-, sex-, and disease-specific information from the Global Burden of Disease 2017 study online data portal (James et al., 2018; Roth et al., 2018) (<http://ghdx.healthdata.org/gbd-results-tool>). Lower- and upper-bounds on all disease burden estimates were also downloaded. Appendix Table S8 summarizes the intervention target disease burden groups. The addressable percentage column represents the percent of the total potential treatment population that is likely to be indicated for a given intervention (*Adding It Up*, 2016; Basu et al., 2013; Denny et al., 2002; Higashi et al., 2015; Higashi et al., 2015; Lamberti et al., 2012; Walker et al., 2013). For example, we assumed that 27% of diarrheal disease cases in under-five children would be addressable by rotavirus vaccine based on available literature on the percent of diarrheal disease caused by rotavirus (Walker et al., 2013).

**Appendix Table S8:** Intervention disease burden targets

Note: TB = Tuberculosis, ART = antiretroviral therapy, HPV = human papillomavirus, IHD = ischemic heart disease.

# Appendix Section 9. Intervention treatment effects

Treatment effects including lower and upper bound (LB, UB) estimates of those effects are based on a review of the published literature (Adam et al., 2005; Bhutta et al., 2014; Davis et al., 2013; “Do Women with Pre-Eclampsia, and Their Babies, Benefit from Magnesium Sulphate?,” 2002; Duley et al., 2010; Fischer Walker & Black, 2011; Higashi et al., 2015; Higashi et al., 2015; Hofmeyr et al., 2014; Law et al., 2009; L. A. Lee et al., 2013; Mandelblatt et al., 2002; Munos et al., 2010; Nadel, 2016; *Reducing Mortality within Universal Health Coverage: The DCP3 Model | DCP3*, 2017; Seid et al., 2018; Siegfried et al., 2009; Stanaway et al., 2018; Theodoratou et al., 2010; Zaidi et al., 2011). Appendix Table S9 summarizes the values and sources of all treatment effects by disease target which were used in our analysis.

**Appendix Table S9:** Treatment effect estimates, by intervention and disease target

Note: TB = Tuberculosis, ART = antiretroviral therapy, HPV = human papillomavirus, IHD = ischemic heart disease.

# Appendix Section 10. Labor and delivery care specific considerations

Throughout this appendix, labor and delivery care is reported as three primary interventions: skilled birth attendance (SBA), basic emergency obstetric care (BEmOC), and comprehensive emergency obstetric care (CEmOC). Each intervention represents a bundle of available interventions when the service is delivered with high quality. Specifically, there are well-defined signal functions used to determine when BEmOC or CEmOC is available to a woman during labor and delivery. Appendix Table S9 summarizes the signal functions and the effect estimates for neonatal and maternal mortality. Neonatal mortality is reported for the bundle as a whole. Similar estimates are not reliably available for the mortality effect of increased coverage of each intervention on maternal outcomes. Instead, we tracked ‘maternal disease burden’ as it relates to specific care outlined by signal functions. However, we always considered SBA, BEmOC, and CEmOC as interventions.

At the aggregate bundle-level, there is some evidence on the impact of both BEmOC and CEmOC on neonatal mortality (Lee et al., 2011). However, similar estimates are not available for the effect of these interventions on maternal mortality. In this analysis, we consider the bundled interventions of BEmOC and CEmOC.

**Appendix Table S10:** Labor and delivery interventions and sub-intervention signal functions

| **Intervention** | | **Neonatal Evidence** | **Maternal Evidence** | **Burden target** | **Included in study** | **Source(s)** |
| --- | --- | --- | --- | --- | --- | --- |
| Skilled Birth Attendance | | | | | | |
| 2 | Presence of skilled health personnel^1^ | X |  | Neonatal disorders | X | (Lee et al., 2011) |
| Basic Emergency Obstetric Care | | | | | | |
| 3 | Full package of BEmOC signal functions | X |  | Neonatal disorders | X | (Lee et al., 2011) |
| 3.1 | Administer parenteral antibiotics |  | X | Maternal sepsis and other maternal infections | X | (Adam et al., 2005) |
| 3.2 | Administer uterotonic drugs (oxytocin) |  | X | Maternal hemorrhage | X | (Magpie Trial, 2002; Duley, 2009) |
| 3.3 | Administer parenteral anticonvulsants for pre-eclampsia and eclampsia |  | X | Maternal hypertensive disorders | X | (Adam et al., 2005) |
|  | Manually remove the placenta | Not included in the analysis for maternal or neonatal mortality effects | | | | |
|  | Remove retained products (e.g. manual vacuum extraction) |  |  |  |  |  |
|  | Preform assisted vaginal delivery |  |  |  |  |  |
| Comprehensive Emergency Obstetric Care | | | | | | |
| 4 | Full package of CEmOC signal functions | X |  | Neonatal disorders | X | (Lee et al., 2011) |
| 4.1 | Perform surgery (e.g. caesarean section) |  | X | Maternal obstructed labor and uterine rupture | X | (Higashi et al., 2015) |
| 4.2 | Perform blood transfusion |  | X | Maternal hemorrhage | X | (Higashi et al., 2015) |

^1^The WHO defines skilled health personnel as “[c]ompetent maternal and newborn health professionals educated, trained and regulated to national and international standards”. Three competencies are included: (1) provide and promote evidence-based care; (2) facilitate the physiological processes during labor and delivery; and (3) identify and manage or refer women and/or newborns with complications (*Definition of Skilled Health Personnel Providing Care during Childbirth the 2018 Joint Statement by WHO, UNFPA, UNICEF, ICM, ICN, FIGO, IPA*, 2018).

# Appendix Section 11. Supplementary results

The below figure plots the same results as Figure 5 in the main paper with both a pessimistic and optimistic uncertainty scenario considering upper and lower bounds on intervention efficacy, the size of the target population, incidence, the case fatality ratio, deaths, and unit costs. All lower and upper bound values are included in the parameter tables exhibited in this appendix with the exception of unit costs. We could not find a consistent source of lower and upper bound estimates for unit costs: instead unit cost lower and upper values were assigned the mean values +/- 50%.

**Appendix Figure S1:** Optimal essential benefit package solution depending on the available budget under a single-objective optimization, with uncertainty (optimistic and pessimistic scenarios) included.

Note: Each panel is a single-objective optimization of either deaths averted (panels A and C) or cases of catastrophic health expenditures (CHE) averted (panels B and D). The auxiliary outcome (CHE cases averted in panel C; deaths averted in panel B) results are plotted based on the optimal benefit package at each budget constraint value but they were not included in the single-objective optimization as an explicit objective or a constraint. Mat. BP screen/treat = Maternal blood pressure screening and treatment with calcium supplementation; SBA = skilled birth attendance.

As for sensitivity analyses using a different catastrophic threshold (25% instead of 10% of consumption expenditures), the level of CHE cases averted at both the 10% and 25% thresholds would remain stable regardless of which measure is used in the optimization (Figure S2).

**Appendix Figure S2:** Optimal essential benefit package solution depending on the available budget under a single-objective optimization to maximize averted cases of catastrophic health expenditures (CHE) (for 10% and 25% catastrophic thresholds) averted.

Lastly, when interventions are bundled by the categories listed in **Appendix Table S1**, incremental budgets can meaningfully improve outcomes. With incremental budget changes, there are sometimes opportunities to fund an entire bundle that was not previously affordable. Given that bundled interventions reflect many real-world decision scenarios, understanding the potential outcomes across a range of budgetary levels is important to identify high-value incremental budget increases for population health and financial risk protection.

**Appendix Figure S3:** Optimal essential health benefit package solution depending on the available budget under a single-objective optimization to maximize: either deaths averted (A/C) or cases of catastrophic health expenditures (CHE) averted (with a 10% catastrophic threshold) (B/D), assuming interventions are purchased as a package by WHO UHC indicator category

# Appendix Section 12. Net present value optimization

Discounting deaths averted and catastrophic health expenditures (CHE) cases averted expected to occur in the future based on the expected time lags listed in Appendix Table S3 does not substantially change the results. The maximum difference in CHE cases averted with discounting compared to the undiscounted estimates is 15 for the pneumococcal conjugate vaccine. The maximum difference in deaths averted comparing discounting to no discounting is 87 for voluntary male medical circumcision.

**Appendix Table S11:** Difference in intervention outcomes when discounting is applied compared to undiscounted estimates

# Appendix Section 13. Additional optimization constraints

This paper focuses on tradeoffs between health and financial risk protection benefits. However, additional constraints could be included that reflect additional priorities for the decision-makers. For instance, if equity (understood as distributional impact) was a concern (e.g. across socioeconomic groups or wealth quintiles), distributional constraints could be incorporated by defining intervention sets for those population subgroups and incorporating constraints to fund such sets. An illustrative example of the constraint formulation is included below, where care for non-communicable disease (NCD) targets is included in the package where I is the vector of interventions considered for inclusion and I_NCD_ is the subset of interventions relevant to NCDs. A constraint is then added that requires that at least one of the interventions in the set S_NCD_ is included (the binary funding indicator is forced to be at least 1). Sequential investments can be enforced by ensuring one intervention is funded before another based on the value of the binary decision variable. Bundled interventions simply require changing the level of decision-making, instead of each intervention being funded individually, a group would be funded altogether.

**Summary of constraint options**

| Equity | $S_{NCD}=I_{NCD}\subseteq I \sum_{i\in S_{NCD}} Z_{i}\geq1$ |
| --- | --- |
|  |  |
| Sequential Investment | $Z_{i}\geq Z_{j}\geq Z_{k}$ |
| Bundled Interventions | Redefine funding decision variables ($Z_{i}^{'}s$) |

# Appendix Section 14. Scenario cases

To estimate optimistic and pessimistic scenarios in our model we considered all combinations of mean, lower, and upper bound estimates for both burden of disease (deaths, incidence, and prevalence) and the intervention disease treatment effects (9 combinations in total: 3 disease burden x 3 treatment effect estimates). When results are presented without uncertainty, those results assume mean disease burden and treatment effect estimates. When upper and lower bound results are included (**Figure 5** in the paper), the lower and upper bounds use the intervention-level high and low deaths averted and catastrophic health expenditure (CHE) cases averted estimates from the 9 possible combinations.

**Appendix Table S12:** Mean, Lower, and Upper Bound Deaths Averted and CHE Cases Averted (10% catastrophic threshold) by Intervention

#
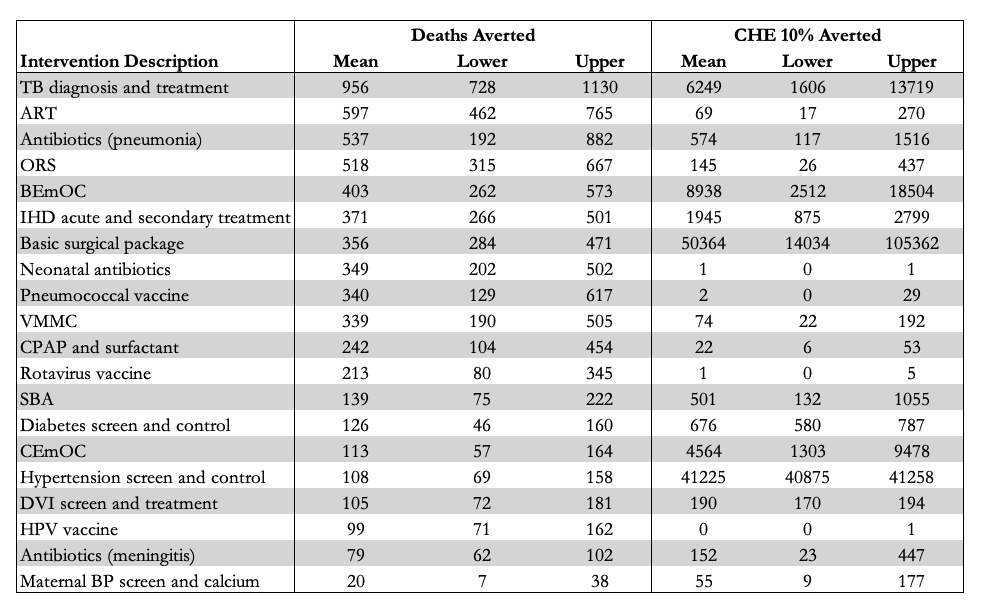
References

Adam, T., Lim, S. S., Mehta, S., Bhutta, Z. A., Fogstad, H., Mathai, M., Zupan, J., & Darmstadt, G. L. (2005). Cost effectiveness analysis of strategies for maternal and neonatal health in developing countries. *BMJ*, *331*(7525), 1107. https://doi.org/10.1136/bmj.331.7525.1107

*Adding It Up: The Costs and Benefits of Investing in Sexual and Reproductive Health 2014—Estimation Methodology*. (2016). Guttmacher Institute. https://www.guttmacher.org/report/adding-it-costs-and-benefits-investing-sexual-and-reproductive-health-2014-methodology

Admasu, K., Haile-Mariam, A., & Bailey, P. (2011). Indicators for availability, utilization, and quality of emergency obstetric care in Ethiopia, 2008. *International Journal of Gynecology & Obstetrics*, *115*(1), 101–105. https://doi.org/10.1016/j.ijgo.2011.07.010

Anh, D. D., Riewpaiboon, A., Tho, L. H., Kim, S. A., Nyambat, B., & Kilgore, P. (2010). Treatment Costs of Pneumonia, Meningitis, Sepsis, and Other Diseases among Hospitalized Children in Viet Nam. *Journal of Health, Population, and Nutrition*, *28*(5), 436–442.

Basu, P., Banerjee, D., Singh, P., Bhattacharya, C., & Biswas, J. (2013). Efficacy and safety of human papillomavirus vaccine for primary prevention of cervical cancer: A review of evidence from phase III trials and national programs. *South Asian Journal of Cancer*, *2*(4), 187–192. https://doi.org/10.4103/2278-330X.119877

Bhutta, Z. A., Das, J. K., Bahl, R., Lawn, J. E., Salam, R. A., Paul, V. K., Sankar, M. J., Blencowe, H., Rizvi, A., Chou, V. B., & Walker, N. (2014). Can available interventions end preventable deaths in mothers, newborn babies, and stillbirths, and at what cost? *The Lancet*, *384*(9940), 347–370. https://doi.org/10.1016/S0140-6736(14)60792-3

Davis, S., Feikin, D., & Johnson, H. L. (2013). The effect of Haemophilus influenzaetype B and pneumococcal conjugate vaccines on childhood meningitis mortality: A systematic review. *BMC Public Health*, *13*(3), S21. https://doi.org/10.1186/1471-2458-13-S3-S21

*Definition of skilled health personnel providing care during childbirth the 2018 joint statement by WHO, UNFPA, UNICEF, ICM, ICN, FIGO, IPA*. (2018). The World Health Organization. https://www.who.int/reproductivehealth/publications/statement-competent-mnh-professionals/en/

Denny, L., Kuhn, L., Pollack, A., & Wright, T. C. (2002). Direct visual inspection for cervical cancer screening. *Cancer*, *94*(6), 1699–1707. https://doi.org/10.1002/cncr.10381

Do women with pre-eclampsia, and their babies, benefit from magnesium sulphate? The Magpie Trial: a randomised placebo-controlled trial. (2002). *The Lancet*, *359*(9321), 1877–1890. https://doi.org/10.1016/S0140-6736(02)08778-0

Duley, L. (2009). The Global Impact of Pre-eclampsia and Eclampsia. *Seminars in Perinatology*, *33*(3), 130–137. https://doi.org/10.1053/j.semperi.2009.02.010

Duley, L., Gülmezoglu, A. M., Henderson-Smart, D. J., & Chou, D. (2010). Magnesium sulphate and other anticonvulsants for women with pre-eclampsia. *The Cochrane Database of Systematic Reviews*, *11*, CD000025. https://doi.org/10.1002/14651858.CD000025.pub2

*Ethiopia Demographic and Health Survey 2016*. (2016). Central Statistical Agency (CSA) [Ethiopia] and ICF. https://dhsprogram.com/pubs/pdf/FR328/FR328.pdf

*Ethiopia Health Accounts, 2013/2014*. (2017). Federal Democratic Republic of Ethiopia Ministry of Health.

*Ethiopia Mini Demographic and Health Survey Key Indicators*. (2019). The DHS Program ICF. https://dhsprogram.com/pubs/pdf/PR120/PR120.pdf

*Ethiopia STEPS Report on Risk Factors for Chronic Non-Communicable Diseases and Prevalence of Selected NCDs*. (2016). Ethiopian Public Health Institute. https://www.who.int/ncds/surveillance/steps/Ethiopia_2015_STEPS_Report.pdf

*Ethiopia’s 5th National Health Accounts*. (2014). https://www.hfgproject.org/wp-content/uploads/2014/04/Ethiopia-NHA-Findings-Briefing-Notes.pdf

*Ethiopia’s NCDI Commission Report*. (2018). http://www.ncdipoverty.org/ethiopia-report

Fischer Walker, C. L., & Black, R. E. (2011). Rotavirus vaccine and diarrhea mortality: Quantifying regional variation in effect size. *BMC Public Health*, *11*(3), S16. https://doi.org/10.1186/1471-2458-11-S3-S16

Higashi, H., Barendregt, J. J., Kassebaum, N. J., Weiser, T. G., Bickler, S. W., & Vos, T. (2015). Surgically avertable burden of obstetric conditions in low- and middle-income regions: A modelled analysis. *BJOG: An International Journal of Obstetrics and Gynaecology*, *122*(2), 228–236. https://doi.org/10.1111/1471-0528.13198

Higashi, Hideki, Barendregt, J. J., Kassebaum, N. J., Weiser, T. G., Bickler, S. W., & Vos, T. (2015). Burden of Injuries Avertable By a Basic Surgical Package in Low- and Middle-Income Regions: A Systematic Analysis From the Global Burden of Disease 2010 Study. *World Journal of Surgery*, *39*, 1–9. https://doi.org/10.1007/s00268-014-2685-x

Hofmeyr, G. J., Lawrie, T. A., Atallah, A. N., Duley, L., & Torloni, M. R. (2014). Calcium supplementation during pregnancy for preventing hypertensive disorders and related problems. *The Cochrane Database of Systematic Reviews*, *6*, CD001059. https://doi.org/10.1002/14651858.CD001059.pub4

Hogan, D. R., Stevens, G. A., Hosseinpoor, A. R., & Boerma, T. (2018). Monitoring universal health coverage within the Sustainable Development Goals: Development and baseline data for an index of essential health services. *The Lancet Global Health*, *6*(2), e152–e168. https://doi.org/10.1016/S2214-109X(17)30472-2

James, S. L., Abate, D., Abate, K. H., Abay, S. M., Abbafati, C., Abbasi, N., Abbastabar, H., Abd-Allah, F., Abdela, J., Abdelalim, A., Abdollahpour, I., Abdulkader, R. S., Abebe, Z., Abera, S. F., Abil, O. Z., Abraha, H. N., Abu-Raddad, L. J., Abu-Rmeileh, N. M. E., Accrombessi, M. M. K., … Murray, C. J. L. (2018). Global, regional, and national incidence, prevalence, and years lived with disability for 354 diseases and injuries for 195 countries and territories, 1990–2017: A systematic analysis for the Global Burden of Disease Study 2017. *The Lancet*, *392*(10159), 1789–1858. https://doi.org/10.1016/S0140-6736(18)32279-7

Lamberti, L. M., Fischer Walker, C. L., & Black, R. E. (2012). Systematic review of diarrhea duration and severity in children and adults in low- and middle-income countries. *BMC Public Health*, *12*, 276. https://doi.org/10.1186/1471-2458-12-276

Law, M. R., Morris, J. K., & Wald, N. J. (2009). Use of blood pressure lowering drugs in the prevention of cardiovascular disease: Meta-analysis of 147 randomised trials in the context of expectations from prospective epidemiological studies. *BMJ (Clinical Research Ed.)*, *338*, b1665. https://doi.org/10.1136/bmj.b1665

Lee, A. C., Cousens, S., Darmstadt, G. L., Blencowe, H., Pattinson, R., Moran, N. F., Hofmeyr, G. J., Haws, R. A., Bhutta, S. Z., & Lawn, J. E. (2011). Care during labor and birth for the prevention of intrapartum-related neonatal deaths: A systematic review and Delphi estimation of mortality effect. *BMC Public Health*, *11*(Suppl 3), S10. https://doi.org/10.1186/1471-2458-11-S3-S10

Lee, L. A., Franzel, L., Atwell, J., Datta, S. D., Friberg, I. K., Goldie, S. J., Reef, S. E., Schwalbe, N., Simons, E., Strebel, P. M., Sweet, S., Suraratdecha, C., Tam, Y., Vynnycky, E., Walker, N., Walker, D. G., & Hansen, P. M. (2013). The estimated mortality impact of vaccinations forecast to be administered during 2011–2020 in 73 countries supported by the GAVI Alliance. *Vaccine*, *31*, B61–B72. https://doi.org/10.1016/j.vaccine.2012.11.035

Mandelblatt, J. S., Lawrence, W. F., Gaffikin, L., Limpahayom, K. K., Lumbiganon, P., Warakamin, S., King, J., Yi, B., Ringers, P., & Blumenthal, P. D. (2002). Costs and benefits of different strategies to screen for cervical cancer in less-developed countries. *Journal of the National Cancer Institute*, *94*(19), 1469–1483. https://doi.org/10.1093/jnci/94.19.1469

Munos, M. K., Walker, C. L. F., & Black, R. E. (2010). The effect of oral rehydration solution and recommended home fluids on diarrhoea mortality. *International Journal of Epidemiology*, *39 Suppl 1*, i75-87. https://doi.org/10.1093/ije/dyq025

Nadel, S. (2016). Treatment of Meningococcal Disease. *The Journal of Adolescent Health: Official Publication of the Society for Adolescent Medicine*, *59*(2 Suppl), S21-28. https://doi.org/10.1016/j.jadohealth.2016.04.013

*OneHealth Tool* (Version 4, 5th edn). (2017). [Computer software]. Avenir Health.

*Reducing Mortality within Universal Health Coverage: The DCP3 Model | DCP3*. (2017). [Working Paper]. Disease Control Priorities Network. http://dcp-3.org/resources/mortality-impact-achieving-essential-universal-health-coverage-low-and-middle-income

Roth, G. A., Abate, D., Abate, K. H., Abay, S. M., Abbafati, C., Abbasi, N., Abbastabar, H., Abd-Allah, F., Abdela, J., Abdelalim, A., Abdollahpour, I., Abdulkader, R. S., Abebe, H. T., Abebe, M., Abebe, Z., Abejie, A. N., Abera, S. F., Abil, O. Z., Abraha, H. N., … Murray, C. J. L. (2018). Global, regional, and national age-sex-specific mortality for 282 causes of death in 195 countries and territories, 1980–2017: A systematic analysis for the Global Burden of Disease Study 2017. *The Lancet*, *392*(10159), 1736–1788. https://doi.org/10.1016/S0140-6736(18)32203-7

Salem, A. B. Z., & Mount, T. D. (1974). A Convenient Descriptive Model of Income Distribution: The Gamma Density. *Econometrica*, *42*(6), 1115–1127. https://doi.org/10.2307/1914221

Seid, M. A., Ayalew, M. B., Muche, E. A., Gebreyohannes, E. A., & Abegaz, T. M. (2018). Drug-susceptible tuberculosis treatment success and associated factors in Ethiopia from 2005 to 2017: A systematic review and meta-analysis. *BMJ Open*, *8*(9), e022111. https://doi.org/10.1136/bmjopen-2018-022111

Siegfried, N., Muller, M., Deeks, J. J., & Volmink, J. (2009). Male circumcision for prevention of heterosexual acquisition of HIV in men. *The Cochrane Database of Systematic Reviews*, *2*, CD003362. https://doi.org/10.1002/14651858.CD003362.pub2

Stanaway, J. D., Afshin, A., Gakidou, E., Lim, S. S., Abate, D., Abate, K. H., Abbafati, C., Abbasi, N., Abbastabar, H., Abd-Allah, F., Abdela, J., Abdelalim, A., Abdollahpour, I., Abdulkader, R. S., Abebe, M., Abebe, Z., Abera, S. F., Abil, O. Z., Abraha, H. N., … Murray, C. J. L. (2018). Global, regional, and national comparative risk assessment of 84 behavioural, environmental and occupational, and metabolic risks or clusters of risks for 195 countries and territories, 1990–2017: A systematic analysis for the Global Burden of Disease Study 2017. *The Lancet*, *392*(10159), 1923–1994. https://doi.org/10.1016/S0140-6736(18)32225-6

The Federal Democratic Republic of Ethiopia. (2018). *The 2015/16 Ethiopian Household Consumption—Expenditure (HCE) Survey* [Statistical Report].

Theodoratou, E., Johnson, S., Jhass, A., Madhi, S. A., Clark, A., Boschi-Pinto, C., Bhopal, S., Rudan, I., & Campbell, H. (2010). The effect of Haemophilus influenzae type b and pneumococcal conjugate vaccines on childhood pneumonia incidence, severe morbidity and mortality. *International Journal of Epidemiology*, *39 Suppl 1*, i172-185. https://doi.org/10.1093/ije/dyq033

Tolla, M. T., Norheim, O. F., Memirie, S. T., Abdisa, S. G., Ababulgu, A., Jerene, D., Bertram, M., Strand, K., Verguet, S., & Johansson, K. A. (2016). Prevention and treatment of cardiovascular disease in Ethiopia: A cost-effectiveness analysis. *Cost Effectiveness and Resource Allocation*, *14*, 10. https://doi.org/10.1186/s12962-016-0059-y

*UNAIDS 2019 estimates*. (2018). UNAIDS. https://aidsinfo.unaids.org/

Walker, C. L. F., Rudan, I., Liu, L., Nair, H., Theodoratou, E., Bhutta, Z. A., O’Brien, K. L., Campbell, H., & Black, R. E. (2013). Global burden of childhood pneumonia and diarrhoea. *The Lancet*, *381*(9875), 1405–1416. https://doi.org/10.1016/S0140-6736(13)60222-6

Watkins, D. A., Qi, J., Kawakatsu, Y., Pickersgill, S. J., Horton, S. E., & Jamison, D. T. (2020). Resource requirements for essential universal health coverage: A modelling study based on findings from Disease Control Priorities, 3rd edition. *The Lancet Global Health*, *8*(6), e829–e839. https://doi.org/10.1016/S2214-109X(20)30121-2

*WHO Tuberculosis profile: Ethiopia (shiny app)* (0.4). (2020). [Computer software]. World Health Organization. https://worldhealthorg.shinyapps.io/tb_profiles/?_inputs_&lan=%22EN%22&iso2=%22ET%22&main_tabs=%22est_tab%22

Zaidi, A. K. M., Ganatra, H. A., Syed, S., Cousens, S., Lee, A. C. C., Black, R., Bhutta, Z. A., & Lawn, J. E. (2011). Effect of case management on neonatal mortality due to sepsis and pneumonia. *BMC Public Health*, *11 Suppl 3*, S13. https://doi.org/10.1186/1471-2458-11-S3-S13
